# Supplementary material for: Principal Component Analysis to Assess the Changes of Yield and Quality in Pinellia ternata at Different Stages after Brassinolide Treatments
Source: Int J Mol Sci. 2022 Dec 6;23(23):15375. doi: 10.3390/ijms232315375 (PMC9740233; doi:10.3390/ijms232315375)
Supplement: Supplementary file 1 [file ijms-23-15375-s001.zip › ijms-2049279-supplementary.pdf]

Supplementary Table S1. The effects of applied BR on active components and soluble protein content in *P. ternata* at different stages

Day	BR concentrations (mg/L)	No.	Total flavone (mg/g DW)		Total alkaloids (mg/g DW)		β-sitosterol (mg/g DW)		Soluble protein（mg/g DW)	
			Tuber	Bulbil	Tuber	Bulbil	Tuber	Bulbil	Tuber	Bulbil
15th	0.00	A0	0.32 ± 0.001 b	0.33 ± 0.004 d	0.50 ± 0.02 c	0.63 ± 0.00 a	1.02 ± 0.01 d	1.15 ± 0.02 ab	9.00 ± 0.12 d	18.30 ± 0.15 d
	0.05	A1	0.28 ± 0.002 d	0.47 ± 0.001 a	0.68 ± 0.00 a	0.41 ± 0.01 b	1.15 ± 0.04 c	1.22 ± 0.02 a	13.09 ± 0.17 b	21.46 ± 0.29 c
	0.10	A2	0.37 ± 0.002 a	0.44 ± 0.001 b	0.67 ± 0.01 a	0.51 ± 0.02 ab	0.98 ± 0.01 d	1.09 ± 0.01 b	13.16 ± 0.05 b	25.12 ± 0.13 a
	0.50	A3	0.29 ± 0.000 c	0.31 ± 0.003 e	0.65 ± 0.00 a	0.56 ± 0.03 a	1.05 ± 0.05 cd	1.11 ± 0.01 b	9.41 ± 0.21 d	23.40 ± 0.12 b
	1 .00	A4	0.37 ± 0.003 a	0.34 ± 0.002 c	0.58 ± 0.01 b	0.55 ± 0.05 a	1.56 ± 0.01 a	1.17 ± 0.02 ab	14.25 ± 0.38 a	23.13 ± 0.25 b
	2 .00	A5	0.32 ± 0.001 b	0.35 ± 0.005 c	0.64 ± 0.01 a	0.59 ± 0.02 a	1.37 ± 0.01 b	0.98 ± 0.02 c	11.50 ± 0.04 c	15.24 ± 0.05 e
45th	0.00	B0	0.28 ± 0.001 bc	0.38 ± 0.001 a	1.22 ± 0.02 ab	0.58 ± 0.01 bc	1.05 ± 0.03 b	1.03 ± 0.02 d	20.39 ± 0.11 a	11.97 ± 0.08 a
	0.05	B1	0.24 ± 0.003 d	0.31 ± 0.003 d	1.32 ± 0.03 a	0.74 ± 0.03 a	1.16 ± 0.03 a	1.87 ± 0.01 a	8.79 ± 0.13 f	11.79 ± 0.09 a
	0.10	B2	0.28 ± 0.002 bc	0.33 ± 0.003 c	1.19 ± 0.02 b	0.63 ± 0.00 b	0.91 ± 0.01 c	1.61 ± 0.04 b	10.80 ± 0.03 e	7.33 ± 0.13 c
	0.50	B3	0.28 ± 0.001 b	0.29 ± 0.001 e	1.22 ± 0.01 ab	0.43 ± 0.01 d	1.06 ± 0.02 ab	1.76 ± 0.03 a	11.46 ± 0.21 d	7.16 ± 0.09 c
	1 .00	B4	0.27 ± 0.001 c	0.35 ± 0.003 b	0.72 ± 0.02 c	0.56 ± 0.01 bc	1.13 ± 0.02 ab	1.61 ± 0.01 b	13.73 ± 0.06 c	10.14 ± 0.11 b
	2 .00	B5	0.33 ± 0.001 a	0.31 ± 0.001 d	0.62 ± 0.03 c	0.55 ± 0.01 c	0.94 ± 0.01 c	1.18 ± 0.04 c	16.89 ± 0.07 b	12.08 ± 0.16 a
60th	0.00	C0	0.28 ± 0.001 c	0.41 ± 0.001 a	1.07 ± 0.02 b	0.66 ± 0.01 e	1.04 ± 0.01 b	1.82 ± 0.03 a	25.77 ± 0.19 d	26.01 ± 0.24 d
	0.05	C1	0.28 ± 0.003 c	0.32 ± 0.001 d	1.17 ± 0.03 a	0.85 ± 0.01 d	0.94 ± 0.04 b	1.44 ± 0.01 b	24.83 ± 0.14 d	29.89 ± 0.06 c
	0.10	C2	0.31 ± 0.002 a	0.34 ± 0.000 c	1.01 ± 0.01 bc	1.45 ± 0.02 b	0.99 ± 0.01 b	0.97 ± 0.01 d	35.74 ± 0.25 a	39.67 ± 0.10 a
	0.50	C3	0.29 ± 0.001 b	0.32 ± 0.001 d	1.05 ± 0.01 bc	1.65 ± 0.03 a	0.96 ± 0.03 b	1.23 ± 0.08 c	33.35 ± 0.00 b	25.13 ± 0.20 e
	1 .00	C4	0.31 ± 0.005 ab	0.30 ± 0.001 e	0.99 ± 0.00 bc	1.15 ± 0.02 c	1.43 ± 0.02 a	1.29 ± 0.00 bc	30.95 ± 0.13 c	24.89 ± 0.19 e
	2 .00	C5	0.32 ± 0.005 a	0.39 ± 0.004 b	0.99 ± 0.01 c	1.11 ± 0.04 c	1.02 ± 0.01 b	1.17 ± 0.02 c	30.69 ± 0.34 c	33.43 ± 0.11 b
75th	0.00	D0	0.29 ± 0.006 b	0.35 ± 0.004 e	1.51 ± 0.01 d	1.37 ± 0.01 d	1.12 ± 0.01 c	2.41 ± 0.04 a	11.88 ± 0.02 e	19.93 ± 0.03 c
	0.05	D1	0.25 ± 0.005 c	0.42 ± 0.005 c	1.70 ± 0.01 b	1.42 ± 0.02 d	1.15 ± 0.01 c	2.14 ± 0.03 bc	19.91 ± 0.26 b	21.37 ± 0.21 b
	0.10	D2	0.35 ± 0.002 a	0.53 ± 0.009 a	1.86 ± 0.00 a	1.51 ± 0.01 bc	1.41 ± 0.04 b	2.34 ± 0.02 ab	16.39 ± 0.03 c	19.14 ± 0.19 d
	0.50	D3	0.34 ± 0.010 a	0.39 ± 0.001 d	1.62 ± 0.02 c	1.65 ± 0.02 a	1.31 ± 0.02 b	1.98 ± 0.02 c	20.12 ± 0.00 b	15.40 ± 0.09 e
	1 .00	D4	0.33 ± 0.011 a	0.48 ± 0.004 b	1.75 ± 0.03 b	1.56 ± 0.01 b	1.13 ± 0.03 c	2.09 ± 0.02 bc	23.84 ± 0.04 a	23.39 ± 0.04 a

	2 .00	D5	0.27 ± 0.003 bc	0.40 ± 0.002 cd	1.72 ± 0.01 b	1.49 ± 0.01 c	1.54 ± 0.00 a	2.23 ± 0.12 abc	14.84 ± 0.03 d	19.17 ± 0.01 d
90th	0.00	E0	0.22 ± 0.003 a	0.32 ± 0.002 a	1.03 ± 0.02 b	1.11 ± 0.00 d	1.25 ± 0.00 ab	1.27 ± 0.02 d	21.86 ± 0.05 c	21.91 ± 0.07 f
	0.05	E1	0.15 ± 0.001 d	0.23 ± 0.002 d	1.09 ± 0.00 b	1.24 ± 0.01 b	1.08 ± 0.02 c	1.61 ± 0.04 c	26.62 ± 0.08 a	24.09 ± 0.05 d
	0.10	E2	0.19 ± 0.000 c	0.29 ± 0.001 b	1.21 ± 0.04 a	1.32 ± 0.00 a	1.15 ± 0.06 bc	1.38 ± 0.06 d	24.56 ± 0.08 b	25.39 ± 0.00 b
	0.50	E3	0.19 ± 0.001 c	0.21 ± 0.001 e	0.91 ± 0.02 c	1.16 ± 0.02 c	1.30 ± 0.02 a	1.56 ± 0.03 c	19.60 ± 0.02 e	22.38 ± 0.10 e
	1 .00	E4	0.15 ± 0.001 d	0.24 ± 0.001 c	0.68 ± 0.00 d	0.99 ± 0.00 e	0.92 ± 0.01 d	2.31 ± 0.01 b	14.18 ± 0.07 f	26.40 ± 0.02 a
	2 .00	E5	0.20 ± 0.001 b	0.24 ± 0.001 c	1.13 ± 0.03 ab	1.16 ± 0.02 c	1.06 ± 0.02 c	2.83 ± 0.04 a	21.24 ± 0.04 d	24.41 ± 0.04 c
105th	0.00	F0	0.29 ± 0.005 a	0.33 ± 0.004 b	1.22 ± 0.02 a	0.87 ± 0.01 d	1.51 ± 0.01 a	1.56 ± 0.03 b	35.87 ± 0.06 d	36.09 ± 0.05 c
	0.05	F1	0.31 ± 0.006 a	0.33 ± 0.002 a	1.25 ± 0.02 a	0.83 ± 0.02 d	1.21 ± 0.01 c	1.44 ± 0.02 bc	39.20 ± 0.04 c	43.32 ± 0.11 a
	0.10	F2	0.24 ± 0.004 c	0.37 ± 0.008 b	0.78 ± 0.03 b	1.04 ± 0.01 c	1.35 ± 0.03 b	1.53 ± 0.04 b	47.06 ± 0.11 a	39.09 ± 0.09 b
	0.50	F3	0.27 ± 0.004 b	0.31 ± 0.003 b	0.44 ± 0.03 c	1.15 ± 0.03 ab	1.22 ± 0.04 c	1.30 ± 0.03 c	40.41 ± 0.33 b	29.62 ± 0.11 f
	1 .00	F4	0.23 ± 0.004 c	0.36 ± 0.004 a	0.69 ± 0.02 b	1.18 ± 0.02 a	1.16 ± 0.03 c	1.31 ± 0.05 c	35.97 ± 0.06 d	34.55 ± 0.05 d
	2 .00	F5	0.25 ± 0.004 bc	0.38 ± 0.004 a	0.68 ± 0.03 b	1.08 ± 0.04 ab	1.12 ± 0.03 c	1.85 ± 0.03 a	40.94 ± 0.09 b	33.32 ± 0.02 e

The different letters are significantly different from each other at same stage ($p < 0.05$). The values are means of four replicates ± SE.

Supplementary Table S2. The effects of applied BR on free amino acid content, soluble sugar content and antioxidant activity in *P. ternata* at different stages

Day	BR concentrations (mg/L)	No.	Free amino acid (g/100 g DW)		Soluble sugar (g/g DW)		Ascorbic acid (mg/g DW)		DPPH radical scavenging activity (%)	
			Tuber	Bulbil	Tuber	Bulbil	Tuber	Bulbil	Tuber	Bulbil
15th	0.00	A0	1.92 ± 0.000 c	1.62 ± 0.003 b	0.199 ± 0.001 c	0.245 ± 0.001 a	0.54 ± 0.005 e	0.73 ± 0.004 e	13.86 ± 0.06 c	15.97 ± 0.06 b
	0.05	A1	2.30 ± 0.003 a	1.49 ± 0.004 c	0.190 ± 0.000 d	0.239 ± 0.001 c	0.51 ± 0.003 f	0.87 ± 0.013 d	12.69 ± 0.07 d	17.90 ± 0.21 a
	0.10	A2	2.12 ± 0.008 b	1.96 ± 0.001 a	0.212 ± 0.001 a	0.243 ± 0.001 ab	0.65 ± 0.000 d	1.02 ± 0.001 c	15.93 ± 0.10 a	17.72 ± 0.32 a
	0.50	A3	0.72 ± 0.007 f	1.45 ± 0.003 d	0.212 ± 0.001 a	0.227 ± 0.000 d	0.68 ± 0.003 c	0.88 ± 0.002 d	16.33 ± 0.19 a	16.44 ± 0.16 b
	1 .00	A4	1.53 ± 0.006 d	0.91 ± 0.000 f	0.208 ± 0.000 b	0.226 ± 0.000 d	0.86 ± 0.010 b	1.33 ± 0.005 a	15.98 ± 0.06 a	18.34 ± 0.21 a
	2 .00	A5	0.82 ± 0.004 e	1.01 ± 0.001 e	0.205 ± 0.000 b	0.241 ± 0.001 bc	0.95 ± 0.003 a	1.18 ± 0.006 b	15.23 ± 0.14 b	15.92 ± 0.07 b
45th	0.00	B0	0.62 ± 0.010 e	0.73 ± 0.001 d	0.248 ± 0.000 a	0.217 ± 0.001 b	0.92 ± 0.000 b	0.89 ± 0.002 b	23.41 ± 0.06 a	14.02 ± 0.13 a
	0.05	B1	0.63 ± 0.003 e	0.61 ± 0.003 e	0.233 ± 0.002 b	0.211 ± 0.001 c	0.88 ± 0.004 c	0.89 ± 0.003 b	9.80 ± 0.17 e	11.90 ± 0.14 c
	0.10	B2	1.43 ± 0.010 a	1.48 ± 0.009 b	0.229 ± 0.001 bc	0.219 ± 0.001 b	0.99 ± 0.008 a	0.84 ± 0.001 d	10.92 ± 0.10 d	12.07 ± 0.09 c
	0.50	B3	0.85 ± 0.013 c	0.70 ± 0.009 d	0.225 ± 0.001 c	0.213 ± 0.002 c	0.93 ± 0.002 b	0.93 ± 0.005 a	10.42 ± 0.03 de	13.94 ± 0.17 ab
	1 .00	B4	0.73 ± 0.006 d	0.86 ± 0.006 c	0.230 ± 0.001 bc	0.213 ± 0.000 c	0.94 ± 0.010 b	0.86 ± 0.001 c	14.11 ± 0.25 c	13.34 ± 0.18 b
	2 .00	B5	1.02 ± 0.011 b	1.95 ± 0.012 a	0.216 ± 0.001 d	0.224 ± 0.000 a	1.02 ± 0.016 a	0.68 ± 0.001 e	16.81 ± 0.34 b	11.21 ± 0.11 d
60th	0.00	C0	0.62 ± 0.019 f	0.62 ± 0.008 e	0.254 ± 0.000 b	0.280 ± 0.001 a	1.26 ± 0.014 c	0.81 ± 0.002 b	7.68 ± 0.04 d	18.10 ± 0.09 a
	0.05	C1	1.91 ± 0.008 b	1.26 ± 0.002 c	0.225 ± 0.002 d	0.265 ± 0.001 b	1.22 ± 0.023 c	0.73 ± 0.003 d	13.03 ± 0.38 a	10.45 ± 0.06 c
	0.10	C2	2.92 ± 0.001 a	3.06 ± 0.013 a	0.235 ± 0.001 c	0.239 ± 0.000 e	1.35 ± 0.007 b	0.74 ± 0.003 d	9.59 ± 0.09 c	10.19 ± 0.14 c
	0.50	C3	0.78 ± 0.009 e	0.69 ± 0.002 d	0.250 ± 0.001 b	0.246 ± 0.002 c	1.54 ± 0.034 a	0.78 ± 0.001 c	11.95 ± 0.11 b	10.52 ± 0.17 c
	1 .00	C4	1.15 ± 0.016 d	0.73 ± 0.016 d	0.253 ± 0.000 b	0.246 ± 0.001 cd	0.82 ± 0.008 d	0.87 ± 0.007 a	11.87 ± 0.03 b	11.57 ± 0.08 b
	2 .00	C5	1.46 ± 0.018 c	2.19 ± 0.003 b	0.268 ± 0.000 a	0.240 ± 0.001 de	0.80 ± 0.002 d	0.81 ± 0.004 b	9.12 ± 0.05 c	8.62 ± 0.17 d
75th	0.00	D0	1.43 ± 0.002 f	1.70 ± 0.003 d	0.209 ± 0.001 a	0.153 ± 0.001 c	0.88 ± 0.003 cd	1.00 ± 0.003 e	12.19 ± 0.12 c	19.61 ± 0.14 c
	0.05	D1	1.82 ± 0.027 d	1.93 ± 0.006 b	0.198 ± 0.001 b	0.149 ± 0.001 d	0.83 ± 0.001 d	0.95 ± 0.005 f	10.27 ± 0.08 d	14.57 ± 0.12 e
	0.10	D2	2.23 ± 0.005 c	1.12 ± 0.008 e	0.135 ± 0.001 c	0.158 ± 0.001 b	0.93 ± 0.006 c	1.55 ± 0.009 b	17.05 ± 0.19 b	23.85 ± 0.14 b
	0.50	D3	2.39 ± 0.000 b	0.69 ± 0.014 f	0.130 ± 0.000 cd	0.185 ± 0.000 a	1.22 ± 0.036 a	1.50 ± 0.003 c	23.34 ± 0.12 a	25.38 ± 0.10 a
	1 .00	D4	2.54 ± 0.004 a	1.86 ± 0.002 c	0.126 ± 0.001 d	0.145 ± 0.001 e	1.02 ± 0.001 b	1.66 ± 0.005 a	12.46 ± 0.13 c	20.47 ± 0.38 c

	2 .00	D5	1.63 ± 0.011 e	1.98 ± 0.002 a	0.195 ± 0.001 b	0.153 ± 0.000 c	0.93 ± 0.005 c	1.31 ± 0.005 d	12.78 ± 0.09 c	16.27 ± 0.16 d
90th	0.00	E0	0.80 ± 0.006 e	1.33 ± 0.002 e	0.079 ± 0.000 c	0.104 ± 0.001 a	0.92 ± 0.016 a	0.60 ± 0.002 ab	12.31 ± 0.27 c	23.04 ± 0.28 a
	0.05	E1	2.67 ± 0.012 a	2.11 ± 0.009 a	0.075 ± 0.001 d	0.085 ± 0.000 d	0.85 ± 0.003 c	0.53 ± 0.001 c	8.63 ± 0.10 e	16.94 ± 0.14 d
	0.10	E2	2.26 ± 0.006 c	0.91 ± 0.001 f	0.089 ± 0.001 a	0.103 ± 0.001 a	0.89 ± 0.005 b	0.62 ± 0.013 a	18.97 ± 0.12 a	21.45 ± 0.16 bc
	0.50	E3	1.41 ± 0.009 d	1.40 ± 0.011 d	0.082 ± 0.001 b	0.095 ± 0.001 b	0.91 ± 0.003 ab	0.57 ± 0.005 b	11.13 ± 0.14 d	22.45 ± 0.27 ab
	1 .00	E4	0.65 ± 0.005 f	1.89 ± 0.003 c	0.077 ± 0.000 cd	0.095 ± 0.000 b	0.58 ± 0.004 d	0.59 ± 0.005 ab	11.60 ± 0.03 d	20.95 ± 0.28 c
	2 .00	E5	2.52 ± 0.007 b	2.07 ± 0.005 b	0.079 ± 0.000 c	0.091 ± 0.001 c	0.61 ± 0.005 d	0.59 ± 0.005 ab	18.16 ± 0.05 b	17.06 ± 0.26 d
105th	0.00	F0	2.97 ± 0.019 b	2.42 ± 0.004 e	0.075 ± 0.001 c	0.086 ± 0.000 b	0.58 ± 0.001 d	0.91 ± 0.015 c	9.09 ± 0.16 a	13.61 ± 0.12 a
	0.05	F1	3.42 ± 0.017 a	2.87 ± 0.008 d	0.081 ± 0.001 ab	0.085 ± 0.000 b	0.62 ± 0.003 c	0.84 ± 0.017 d	7.90 ± 0.10 bc	11.96 ± 0.13 bc
	0.10	F2	2.85 ± 0.004 c	3.57 ± 0.004 a	0.083 ± 0.000 a	0.095 ± 0.000 a	0.64 ± 0.003 bc	0.98 ± 0.011 ab	8.54 ± 0.10 ab	13.17 ± 0.10 a
	0.50	F3	2.93 ± 0.013 bc	2.42 ± 0.010 e	0.081 ± 0.000 b	0.094 ± 0.000 a	0.56 ± 0.003 d	0.95 ± 0.004 bc	8.97 ± 0.09 a	12.08 ± 0.23 b
	1 .00	F4	2.00 ± 0.033 e	3.27 ± 0.017 b	0.068 ± 0.001 d	0.095 ± 0.001 a	0.65 ± 0.010 b	0.93 ± 0.005 bc	7.30 ± 0.30 c	13.48 ± 0.10 a
	2 .00	F5	2.56 ± 0.020 d	3.01 ± 0.018 c	0.080 ± 0.001 b	0.077 ± 0.000 c	0.70 ± 0.007 a	1.03 ± 0.011 a	7.85 ± 0.16 bc	11.32 ± 0.10 c

The different letters are significantly different from each other at same stage ($p < 0.05$). The values are means of four replicates ± SE.
